# Pan-cancer analysis of forkhead box Q1 as a potential prognostic and immunological biomarker

**DOI:** 10.3389/fgene.2022.944970

**Published:** 2022-09-01

**Authors:** Qiguan Dong, Lirong Yan, Qingbang Xu, Xianliang Hu, Yan Yang, Ruiwu Zhu, Qian Xu, Yuchao Yang, Bengang Wang

**Affiliations:** ^1^ Department of Radiation Oncology, General Hospital of Fushun Mining Bureau of Liaoning Health Industry Group, Fushun, China; ^2^ Tumour Etiology and Screening Department of Cancer Institute and General Surgery, Liaoning Provincial Education Department, Key Laboratory of Cancer Etiology and Prevention, The First Affiliated Hospital of China Medical University, China Medical University, Shenyang, China; ^3^ Department of Pain Medicine, Union Hospital, Tongji Medical College, Huazhong University of Science and Technology, Wuhan, China; ^4^ Department of Breast Surgery, The 3rd People’s Hospital of Liaoyang, Liaoyang, China; ^5^ Department of Thoracic Surgery, General Hospital of Fushun Mining Bureau of Liaoning Health Industry Group, Fushun, China; ^6^ Department of Neurology, General Hospital of Fushun Mining Bureau of Liaoning Health Industry Group, Fushun, China; ^7^ Department of Hepatobiliary Surgery, Institute of General Surgery, First Hospital of China Medical University, Shenyang, China

**Keywords:** FOXQ1, prognosis, tumor mutational burden, microsatellite instability, tumor microenvironment, immune cell infiltration, pan-cancer

## Abstract

Forkhead box Q1 (FOXQ1) is a member of the forkhead transcription factor family involved in the occurrence and development of different tumors. However, the specific expression patterns and functions of FOXQ1 in pan-cancer remain unclear. Therefore, we collected the expression, mutation, and clinical information data of 33 tumors from The Cancer Genome Atlas database. Via public pan-cancer transcriptome data analysis, we found that FOXQ1 is differentially expressed in various tumors at tissue and cell levels, such as liver hepatocellular carcinoma, colon adenocarcinoma, lung adenocarcinoma, lung squamous cell carcinoma, thyroid carcinoma, and kidney renal clear cell carcinoma. Kaplan–Meier and Cox analyses suggested that FOXQ1 expression was associated with poor overall survival of cutaneous melanoma and thymoma. Its expression was also associated with good disease-specific survival (DSS) in prostate adenocarcinoma but poor DSS in liver hepatocellular carcinoma. In addition, FOXQ1 expression was associated with poor disease-free survival of pancreatic adenocarcinoma. Moreover, FOXQ1 expression was closely related to the tumor mutational burden in 14 tumor types and microsatellite instability (MSI) in 8 tumor types. With an increase in stromal and immune cells, FOXQ1 expression was increased in breast invasive carcinoma, pancreatic adenocarcinoma, thyroid carcinoma, lung adenocarcinoma, and ovarian serous cystadenocarcinoma, while its expression was decreased in pancreatic adenocarcinoma, bladder urothelial carcinoma, and stomach adenocarcinoma. We also found that FOXQ1 expression was related to the infiltration of 22 immune cell types in different tumors (*p* < 0.05), such as resting mast cells and resting memory CD4 T cells. Last, FOXQ1 was coexpressed with 47 immune-related genes in pan-cancer (*p* < 0.05). In conclusion, FOXQ1 expression is closely related to prognosis, clinicopathological parameters, cancer-related pathway activity, the tumor mutational burden, MSI, the tumor microenvironment, immune cell infiltration, and immune-related genes and has the potential to be a diagnostic and prognostic biomarker as well as an immunotherapy target for tumors. Our findings provide important clues for further mechanistic research into FOXQ1.

## Introduction

Cancer is a major public health problem and the leading cause of death worldwide (Bray et al., 2018; Siegel et al., 2020). Because of a lack of breakthroughs in tumor treatment, it has become the biggest obstacle to improving human life expectancy. Therefore, it is vitally important to explore the etiology and pathological mechanism of tumors to develop effective therapeutic regimens.

Studies have found that tumors occurring in different organs but of the same histological type have strong molecular similarities, such as head and neck, lung, esophageal, bladder, and cervical cancers (Shen et al., 2020). There are also molecular similarities in tumors whose anatomical structures belong to the same system, such as gastric, colon, and rectal cancers (Shen et al., 2020). Therefore, exploration of the phenotypic characteristics of molecules in pan-cancer will help to elucidate their commonalities in tumors and their internal regulatory mechanisms.

Forkhead box Q1 (FOXQ1), also called HNF-3/fkh homolog-1, is a member of the forkhead box protein family. As a transcription factor, FOXQ1 encodes multiple functional amino acid proteins, activates T cells and autoimmunity, promotes epithelial differentiation, inhibits smooth muscle cell differentiation, and regulates mucous protein expression and particle concentration in gastric surface mucous cells (Hoggatt et al., 2000; Jonsson and Peng, 2005; Hannenhalli and Kaestner, 2009). In addition, studies have shown that FOXQ1 protein promotes tumor angiogenesis (Peng et al., 2015). Knockdown of FOXQ1 expression suppresses the angiogenic capacity of tumor cells by regulating VEGF, which is an activator of angiogenesis that is secreted by tumor cells (Ellis and Hicklin, 2008; Christensen et al., 2013; Li et al., 2016). FOXQ1 alters the tumor microenvironment (TME) by regulating versican V1. Researchers have confirmed that versican V1, which promotes tumor cell metastasis and macrophage recruitment, is a direct transcriptional target of FOXQ1. Versican V1 overexpression regulates FOXQ1 and induces tumor cells to secrete chemokine ligand 2, which is able to increase the numbers of tumor-associated macrophages, whereas the inhibition of versican V1 can significantly inhibit FOXQ1 expression (Qian and Pollard, 2010; Xia et al., 2014; Li et al., 2016). Research suggests that FOXQ1 is closely related to the occurrence of different cancers and could accelerate the migration of esophageal, gastric, and colorectal cancer cells (Pei et al., 2015; Zhang et al., 2016; Liu et al., 2017). FOXQ1 can regulate other biological behaviors of tumors, such as invasion, apoptosis, and epithelial–mesenchymal transition (Qiao et al., 2011; Li et al., 2016). The evidence indicates a correlation of FOXQ1 with tumor progression, and a deepening of our understanding of FOXQ1 could provide important clues for research into pathogenic tumor mechanisms.

FOXQ1 can regulate the immune response and influence tumor progression (Qian and Pollard, 2010; Xia et al., 2014; Li et al., 2016). The expression profile of FOXQ1 in pan-cancer is unclear, the intrinsic correlation of structural variation with FOXQ1 expression is unknown, and whether its effect on tumor progression is related to changes in immune function remains to be elucidated. Resolution of the above key problems could help to deepen the systemic recognition of FOXQ1 in pan-cancer and lay a solid foundation for subsequent mechanistic research.

In this study, The Cancer Genome Atlas (TCGA), Cancer Cell Line Encyclopedia (CCLE), and Oncomine databases were used to analyze the expression profile of FOXQ1 at different levels and the correlation of its expression with prognosis, clinicopathological parameters, cancer-related pathways, tumor mutational burden (TMB), microsatellite instability (MSI), the TME, immune cell infiltration, and immune-related genes to comprehensively unravel the expression profile of FOXQ1 in pan-cancer; understand any changes in expression-related characteristics; and illustrate potential pathogenic mechanisms. Our goal was to provide a reliable basis for identifying potential diagnostic and prognostic biomarkers as well as anticancer immunotherapy targets.

## Materials and methods

### Comprehensive analysis of the forkhead box Q1 expression profile in pan-cancer

UCSC Xena (https://xenabrowser.net/) was used to download expression, mutation, and clinical information data on 33 tumors. The expression data were normalized by fragments per kilobase of exon model per million mapped fragments. The differential expression of FOXQ1 was identified by a Wilcoxon rank-sum test (*p* < 0.05). All analyses and visualizations were performed with RStudio 3.6.1. A box diagram was further designed using the “ggpubr” R package. The Oncomine database (https://www.oncomine.org/) was used to validate any differential results with TCGA, and the cutoff criteria were considered to be |log_2_FC|≥2, *p* < 0.05, and top 10% gene rank. The online website CCLE (https://portals.broadinstitute.org/ccle/) was used to detect FOXQ1 expression in 1,057 cell lines from 36 tumors. Tumors were named as follows: ACC: adrenocortical carcinoma; BLCA: bladder urothelial carcinoma; BRCA: breast invasive carcinoma; CESC: cervical squamous cell carcinoma and endocervical adenocarcinoma; CHOL: cholangiocarcinoma; COAD: colon adenocarcinoma; DLBC: lymphoid neoplasm diffuse large B-cell; ESCA: esophageal carcinoma; GBM: glioblastoma multiforme; HNSC: head and neck squamous cell carcinoma; KICH: kidney chromophobe; KIRC: kidney renal clear cell carcinoma; KIRP: kidney renal papillary cell carcinoma; LAML: acute myeloid leukemia; LGG: brain lower-grade glioma; LIHC: liver hepatocellular carcinoma; LUAD: lung adenocarcinoma; LUSC: lung squamous cell carcinoma; MESO: mesothelioma; OV: ovarian serous cystadenocarcinoma; PAAD: pancreatic adenocarcinoma; PCPG: pheochromocytoma and paraganglioma; PRAD: prostate adenocarcinoma; READ: rectum adenocarcinoma; SARC: sarcoma; SKCM: skin cutaneous melanoma; STAD: stomach adenocarcinoma; TGCT: testicular germ cell tumors; THCA: thyroid carcinoma; THYM: thymoma; UCEC: uterine corpus endometrial carcinoma; and UCS: uterine carcinosarcoma; UVM: uveal melanoma.

### Correlation analysis of forkhead box Q1 expression with prognosis in pan-cancer

Survival data in TCGA were used to evaluate the association of FOXQ1 expression with prognosis in different tumor patients. For survival curve analysis, all tumor patients were divided into two groups according to the median FOXQ1 expression. The Kaplan–Meier method and log-rank test were used to analyze the influence of FOXQ1 expression on overall survival (OS), disease-specific survival (DSS), disease-free survival (DFS), and progression-free survival (PFS) (*p* < 0.05), and a survival curve was delineated using the “survminer” and “survival” R packages (Miao et al., 2020). OS refers to the period of time between initial diagnosis and death (any cause). Here, DSS is defined as the length of time from the initial diagnosis to death from the type of cancer diagnosed. DFS refers to the time period between the date of diagnosis and the first new event of tumor progression (after initial diagnosis and treatment) after the patient’s disease-free state. New events may be associated with local recurrence, distant metastasis, development of a new primary tumor (in the same organ), or death due to progression of the same tumor. PFS refers to the period from the date of diagnosis to the date of the first new tumor event, including disease progression, local recurrence, distant metastasis, new primary tumor, or death due to a tumor (Liu et al., 2018). Then, Cox analysis was applied for the abovementioned prognostic analysis (*p* < 0.05). The calculation and visualization were performed using the “Survival” and “forestplot” R packages. The sample size for the prognosis analysis is listed in Supplementary Table S1.

### Correlation analysis of forkhead box Q1 expression with clinicopathological parameters in pan-cancer

Clinical information data in TCGA were used to evaluate the relationship of FOXQ1 expression with age and pan-cancer stage and grade. Patients were divided into two groups using 65 years as a cutoff value to evaluate the relationship of FOXQ1 expression with age in different tumors. According to the clinical pathological stage, the patients were divided into two groups—I + II and III + IV—to analyze the influence of FOXQ1 expression on the stage. According to the clinical pathological grade, the patients were divided into two groups—G1 + G2 and G3 + G4—to analyze the relationship between FOXQ1 expression and pathological grade. The clinicopathological correlation analysis was mainly performed using the “limma” and “ggpubr” R packages. All analyses were performed using RStudio 3.6.1; *p <* 0.05 was considered significant.

### Gene set enrichment analysis of forkhead box Q1 expression in pan-cancer

Cancer hallmark gene sets from MSigDB collections, which summarize and represent specific well-defined biological states or processes, were applied in Gene Set Enrichment Analysis (Liberzon et al., 2015). Spearman correlation analysis of FOXQ1 expression with cancer hallmark pathway activation was performed using the “limma,” “org.Hs.eg.db,” “clusterProfiler,” and “enrichplot” R packages. *p* < 0.05 was regarded as the cutoff value.

### Correlation analysis of forkhead box Q1 expression with the tumor mutational burden and MSI in pan-cancer

Mutation data in TCGA were used to assess the TMB and MSI of tumors. TMB was defined as the number of bases mutated per million bases. The MSI score was derived from TCGA data. Next, Spearman correlation analysis was used to assess the association of FOXQ1 expression with the TMB and MSI (*p* < 0.05) using the “cor.test” command. The visualization was performed with the “fmsb” R package.

### Correlation analysis of forkhead box Q1 expression with the TME and immune cell infiltration in pan-cancer

The ESTIMATE algorithm in the “estimate” and “limma” R packages was used to analyze TCGA expression data and thereby calculate the immune score and stromal score. CIBERSORT was used to calculate the immune cell infiltration score. Next, the correlation of FOXQ1 expression with the TME and immune cell infiltration was performed with Spearman correlation analysis (*p <* 0.05) using the “ggplot2,” “ggpubr” and “ggExtra” R packages. In addition, TIMER was used to analyze the association of FOXQ1 with immune cell infiltration (http://timer.comp-genomics.org/).

### Coexpression analysis of FOXQ1 expression with immune-related genes in pan-cancer

In total, 47 immune-related genes were summarized after the literature review, and then TCGA expression data were used for the coexpression of FOXQ1 and immune-related genes. The coexpression profile of FOXQ1 with immune-related genes was evaluated using the “limma” package (*p <* 0.05). The “reshape2” and “RColorBrewer” packages were used to perform visualization.

## Results

### Identification of the forkhead box Q1 expression profile in pan-cancer

#### Differential expression of forkhead box Q1 at the tissue level in pan-cancer

**FIGURE 1:**
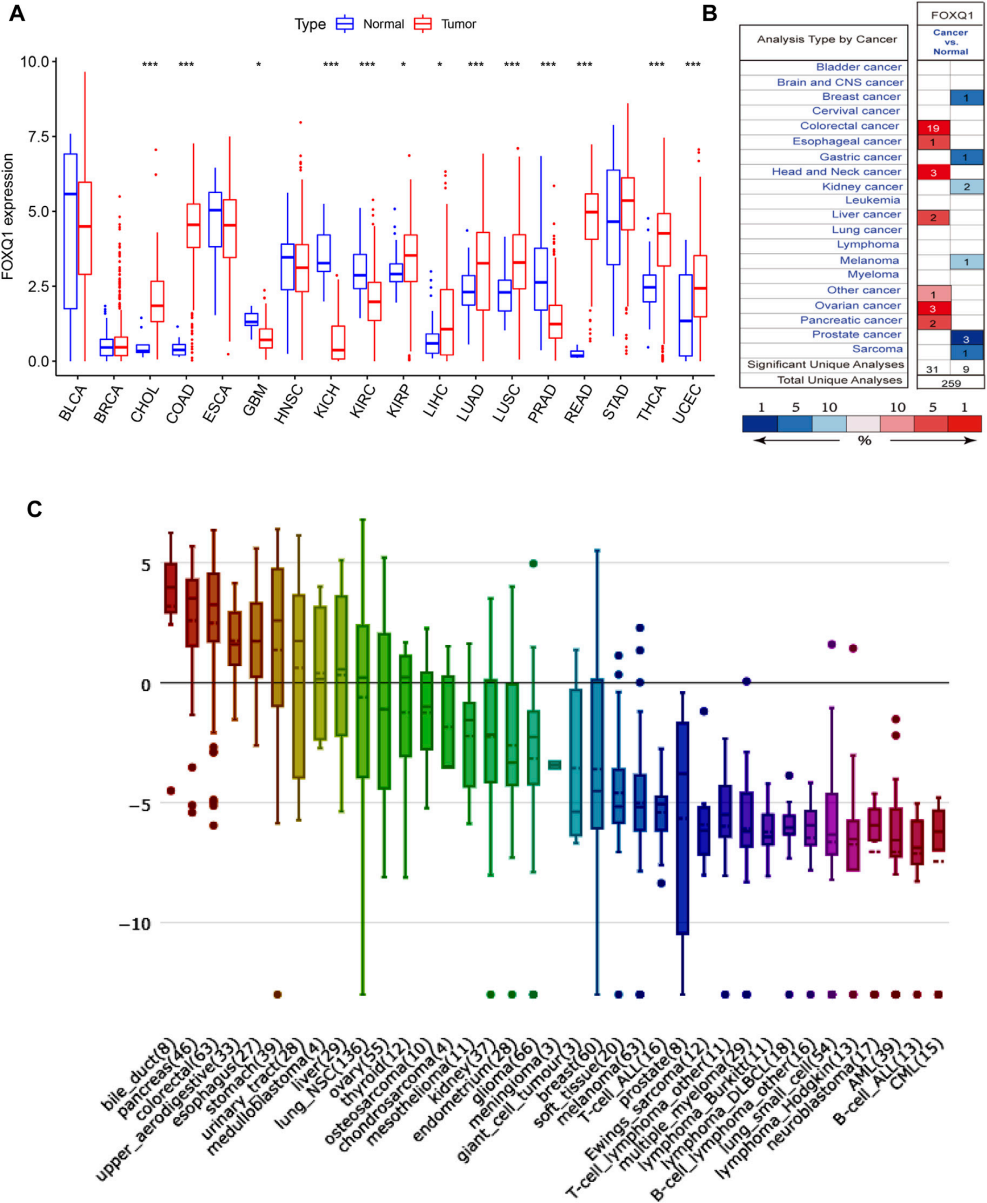
FOXQ1 expression profiles at tissue and cell levels in pan-cancer. **(A)** The expression profile of FOXQ1 at tissue level in TCGA database. Red color represents tumor samples and blue color represents normal samples. **(B)** The expression profile of FOXQ1 at tissue level in Oncomine database. The number represent the count of datasets in the plot. Red color represents high expression and blue color represents low expression. The color depth was related to the sequencing of gene expression differences. Red saturated color represented top 1%. Medium saturated color block represents top 5%; White blocks represent the top 10%, blue and so on. The “Total Unique Analyses” means that the total number of datasets for differential analysis. **(C)** The expression profile of FOXQ1 at cell level in CCLE database. The numbers in brackets represent the sample size of tumor cell lines and the y axis represent the expression level of FOXQ1. Red, green, blue, and violet indicate a relative decrease in expression levels. TCGA, The Cancer Genome Atlas; CCLE, Cancer Cell Line Encyclopedia.

The sample size selected per cancer type is listed in Supplementary Table S2. Differential analysis in TCGA indicated that compared with normal tissues, FOXQ1 was upregulated in cholangiocarcinoma, colon adenocarcinoma, kidney renal papillary cell carcinoma, liver hepatocellular carcinoma, lung adenocarcinoma, lung squamous cell carcinoma, rectum adenocarcinoma, thyroid carcinoma, and uterine corpus endometrial carcinoma and downregulated in glioblastoma multiforme, kidney chromophobe, kidney renal clear cell carcinoma, and prostate adenocarcinoma (Figure 1A). The validation results of Oncomine suggested that FOXQ1 was upregulated in colon adenocarcinoma and liver hepatocellular carcinoma and downregulated in kidney cancer and prostate adenocarcinoma, which was consistent with TCGA results (Figure 1B).

#### Differential expression of forkhead box Q1 at the cell level in pan-cancer

The RNA-seq data of CCLE was used to detect FOXQ1 expression in 1,057 cell lines from 36 tumors. The results demonstrated that the FOXQ1 expression level was high in pancreatic adenocarcinoma, liver hepatocellular carcinoma, colon adenocarcinoma, lung cancer, and thyroid carcinoma but low in other tumors, such as kidney cancer, breast invasive carcinoma, uterine corpus endometrial carcinoma, cholangiocarcinoma, and sarcoma (Figure 1C).

### Correlation between forkhead box Q1 expression and prognosis in pan-cancer

#### Association of forkhead box Q1 expression with overall survival

**FIGURE 2:**
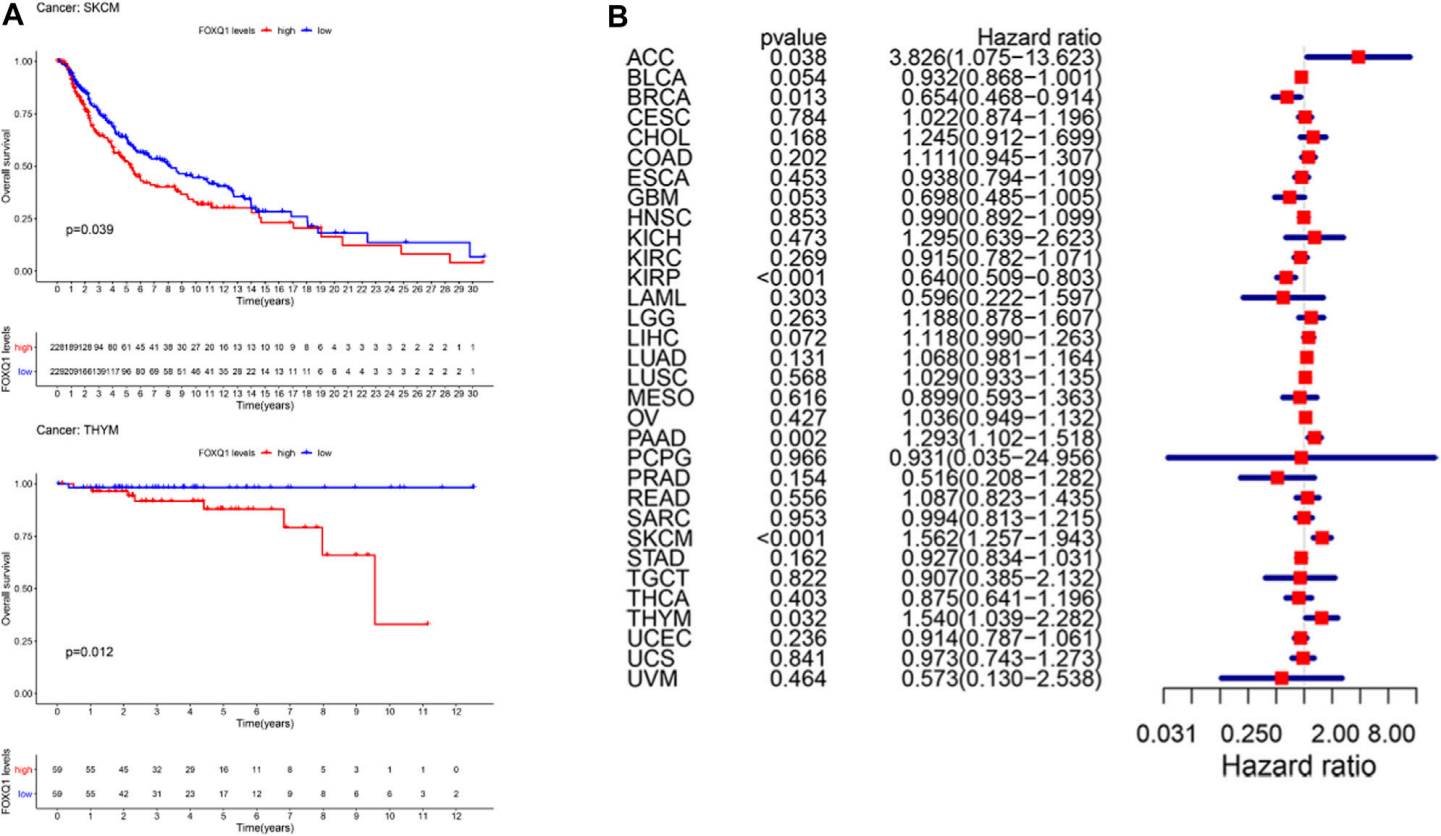
Correlation of forkhead box Q1 (FOXQ1) expression with overall survival in different cancer types. **(A)** Kaplan–Meier curve based FOXQ1 expression in cancer. Red color represents high expression and blue color represents low expression. **(B)** forest plot for the overall survival analysis of FOXQ1 in pan-cancer.

The correlation of FOXQ1 expression with OS was analyzed. The median expression of FOXQ1 was regarded as the cutoff value for dividing patients into two groups. A Kaplan–Meier cumulative survival curve indicated that FOXQ1 expression was associated with unfavorable prognosis in skin cutaneous melanoma and thymoma (Figure 2A). Furthermore, Cox regression analysis indicated that FOXQ1 expression was associated with better prognosis in breast invasive carcinoma and kidney renal papillary cell carcinoma but with worse prognosis in cholangiocarcinoma, thymoma, pancreatic adenocarcinoma, and skin cutaneous melanoma (Figure 2B).

#### Association of forkhead box Q1 expression with disease-specific survival in pan-cancer

**FIGURE 3:**
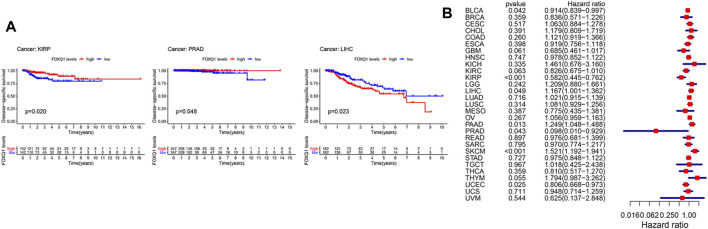
Association of forkhead box Q1 (FOXQ1) expression with disease-specific survival across different cancer types. **(A)** Kaplan–Meier curve based FOXQ1 expression in cancer. The red color represents high expression, and the blue color represents low expression. **(B)** forest plot for the disease-specific survival analysis of FOXQ1 in pan-cancer.

In addition, we used a Kaplan–Meier cumulative survival curve to analyze the relationship between FOXQ1 expression with DSS. FOXQ1 expression was correlated with good DSS of tumor patients in kidney renal clear cell carcinoma and prostate adenocarcinoma and associated with poor DSS in liver hepatocellular carcinoma (Figure 3A). Cox regression analysis indicated that FOXQ1 expression suggested good prognosis in bladder urothelial carcinoma, kidney renal papillary cell carcinoma, prostate adenocarcinoma, and uterine corpus endometrial carcinoma and poor prognosis in liver hepatocellular carcinoma, pancreatic adenocarcinoma, and skin cutaneous melanoma (Figure 3B).

#### Association of forkhead box Q1 expression with disease-free survival in pan-cancer

**FIGURE 4:**
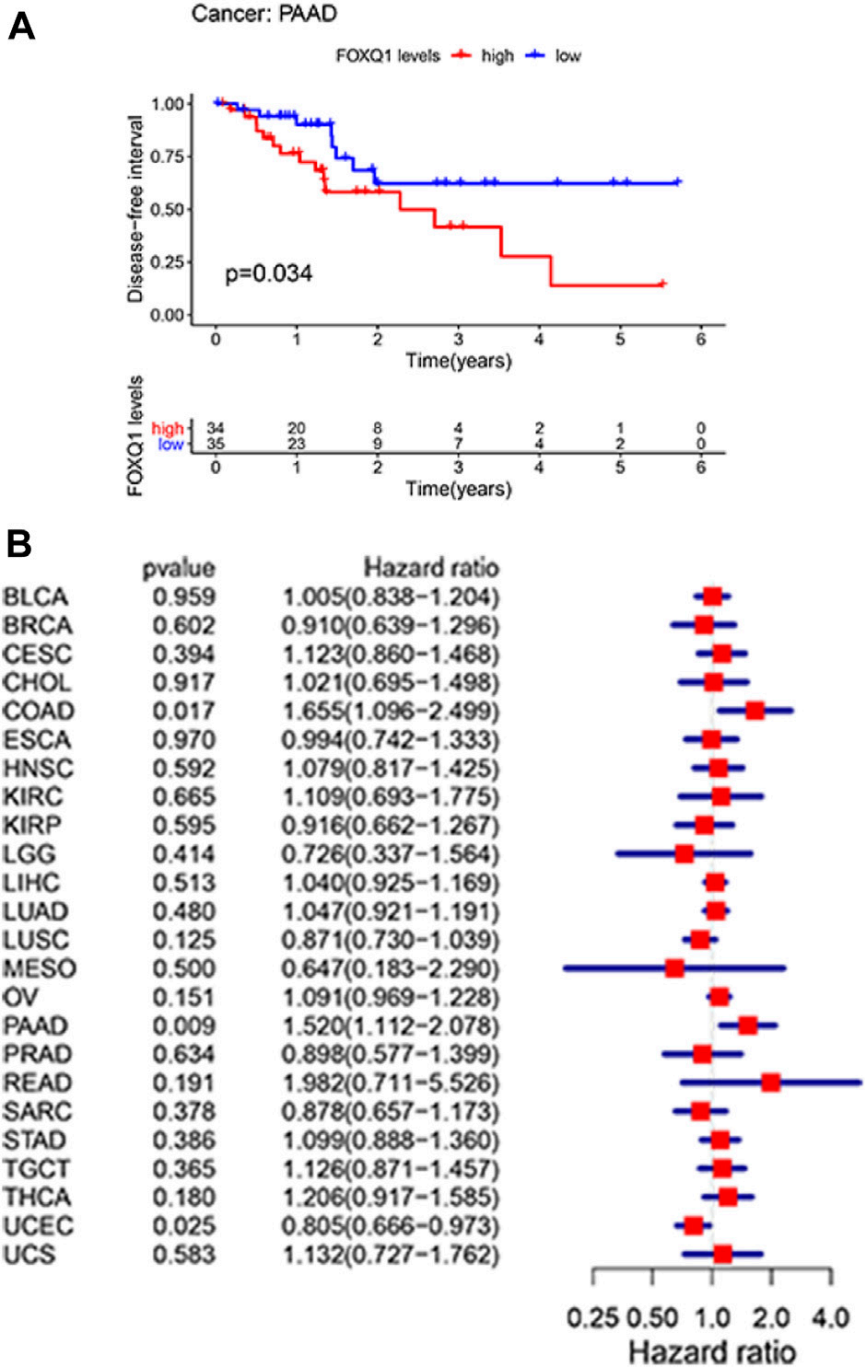
Correlation of forkhead box Q1 (FOXQ1) expression with disease-free survival in pan-cancer. **(A)** Kaplan–Meier curve based FOXQ1 expression in cancer. The red color represents high expression, and the blue color represents low expression. **(B)** forest plot for the disease-free survival of FOXQ1 in pan-cancer.

A Kaplan–Meier cumulative survival curve was next applied to explore the correlation of FOXQ1 expression with DFS. The findings suggested that FOXQ1 expression was related to poor DFS in pancreatic adenocarcinoma (Figure 4A). Cox regression analysis demonstrated that FOXQ1 expression was related to good DFS in uterine corpus endometrial carcinoma and poor DFS in colon adenocarcinoma and pancreatic adenocarcinoma (Figure 4B).

#### Association of forkhead box Q1 expression with progression-free survival in pan-cancer

**FIGURE 5:**
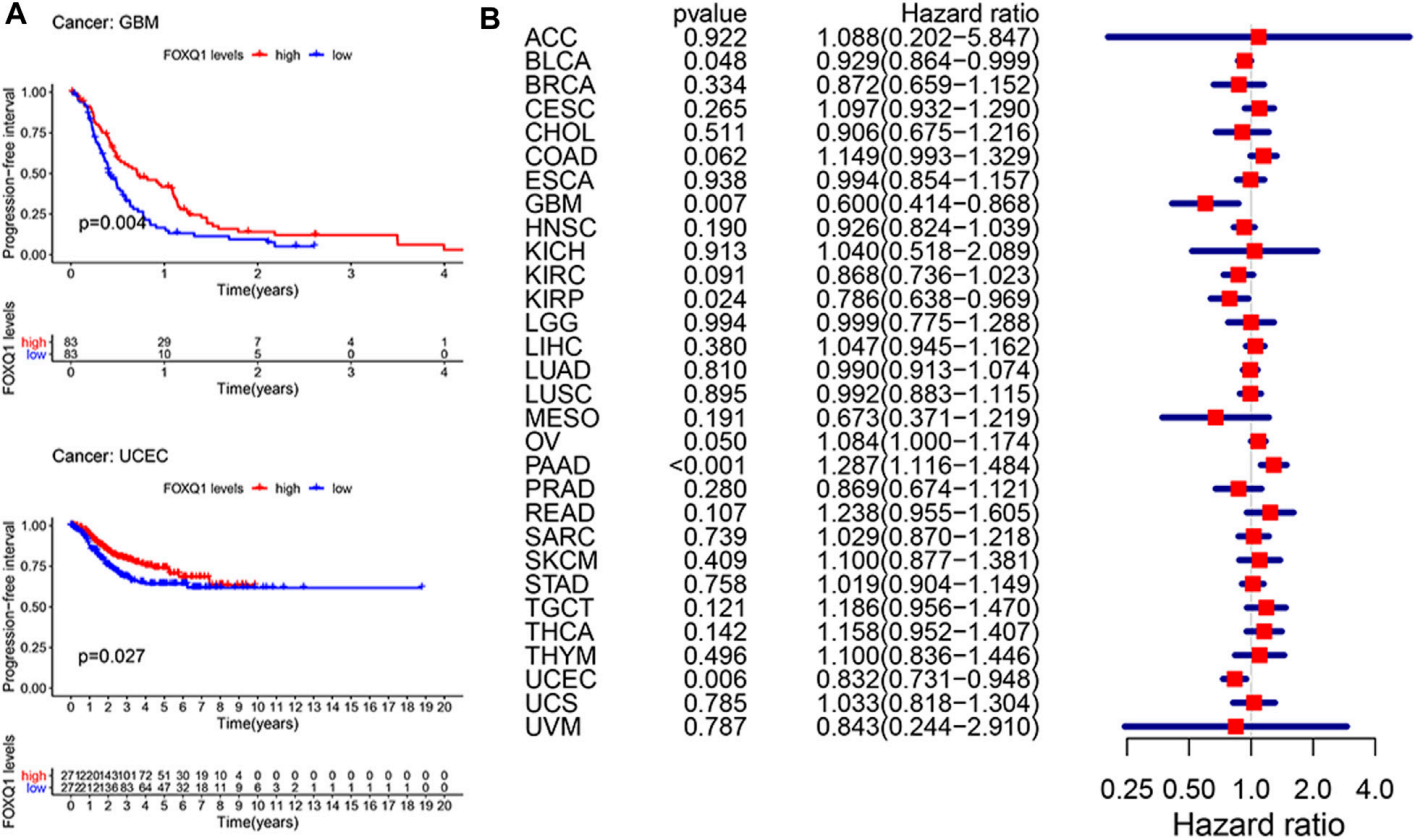
Correlation of forkhead box Q1 (FOXQ1) expression with progress-free survival in pan-cancer. **(A)** Kaplan–Meier curve based FOXQ1 expression in cancer. The red color represents high expression, and the blue color represents low expression. **(B)** forest plot for the progress-free survival analysis of FOXQ1 in pan-cancer.

Last, a Kaplan–Meier cumulative survival curve was used to explore the correlation of FOXQ1 expression with PFS. FOXQ1 expression was related to a favorable PFS in glioblastoma multiforme and corpus endometrial carcinoma (Figure 5A). Cox regression analysis demonstrated that FOXQ1 expression was associated with a benign PFS in uterine corpus endometrial carcinoma, kidney renal papillary cell carcinoma, and glioblastoma multiforme and a poor PFS in pancreatic adenocarcinoma (Figure 5B).

### Correlation between forkhead box Q1 expression and clinicopathological characteristics in pan-cancer

**FIGURE 6:**
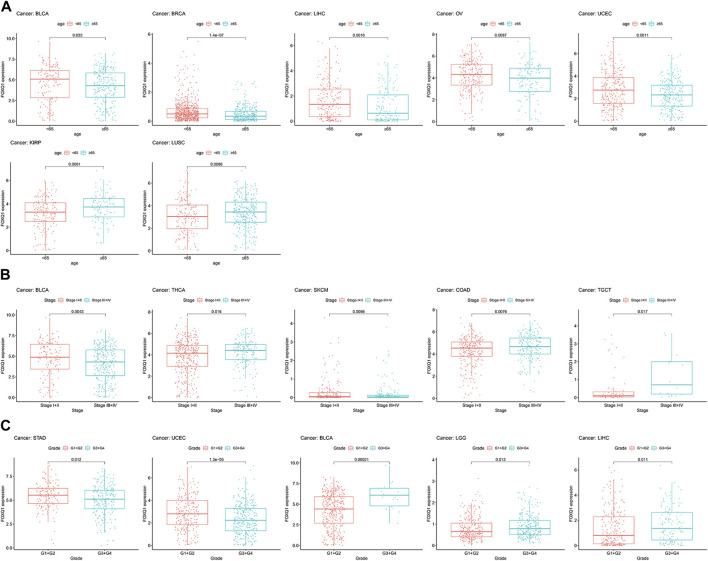
Correlation between forkhead box Q1 (FOXQ1) expression and clinical pathological parameters in pan-cancer. **(A)** association of FOXQ1 expression with age. The red color represents <65 years, and the blue color represents ≥65 years. **(B)** association of FOXQ1 expression with stage. The red color represents the I + II stage, and the blue color represents the III + IV stage. **(C)** association of FOXQ1 expression with grade. The red color represents G1 + G2, and the blue color represents G3 + G4.

Next, we analyzed the association of FOXQ1 expression with clinicopathological characteristics. Compared with patients ≥65 years of age, the FOXQ1 expression level in patients <65 years of age was higher in bladder urothelial carcinoma, breast invasive carcinoma, liver hepatocellular carcinoma, ovarian serous cystadenocarcinoma, and uterine corpus endometrial carcinoma and lower in kidney renal papillary cell carcinoma and lung squamous cell carcinoma (Figure 6A). Compared with the III + IV stage, the FOXQ1 expression level in the I + II stage was higher in bladder urothelial carcinoma and skin cutaneous melanoma and lower in rectum adenocarcinoma, thyroid carcinoma, and testicular germ cell tumors (Figure 6B). Compared with the G1 + G2 grade, the FOXQ1 expression level in the G3 + G4 grade was higher in bladder urothelial carcinoma, brain lower-grade glioma, and liver hepatocellular carcinoma but lower in stomach adenocarcinoma and uterine corpus endometrial carcinoma (Figure 6C).

#### Association of forkhead box Q1 expression with cancer-related pathways in pan-cancer

**FIGURE 7:**
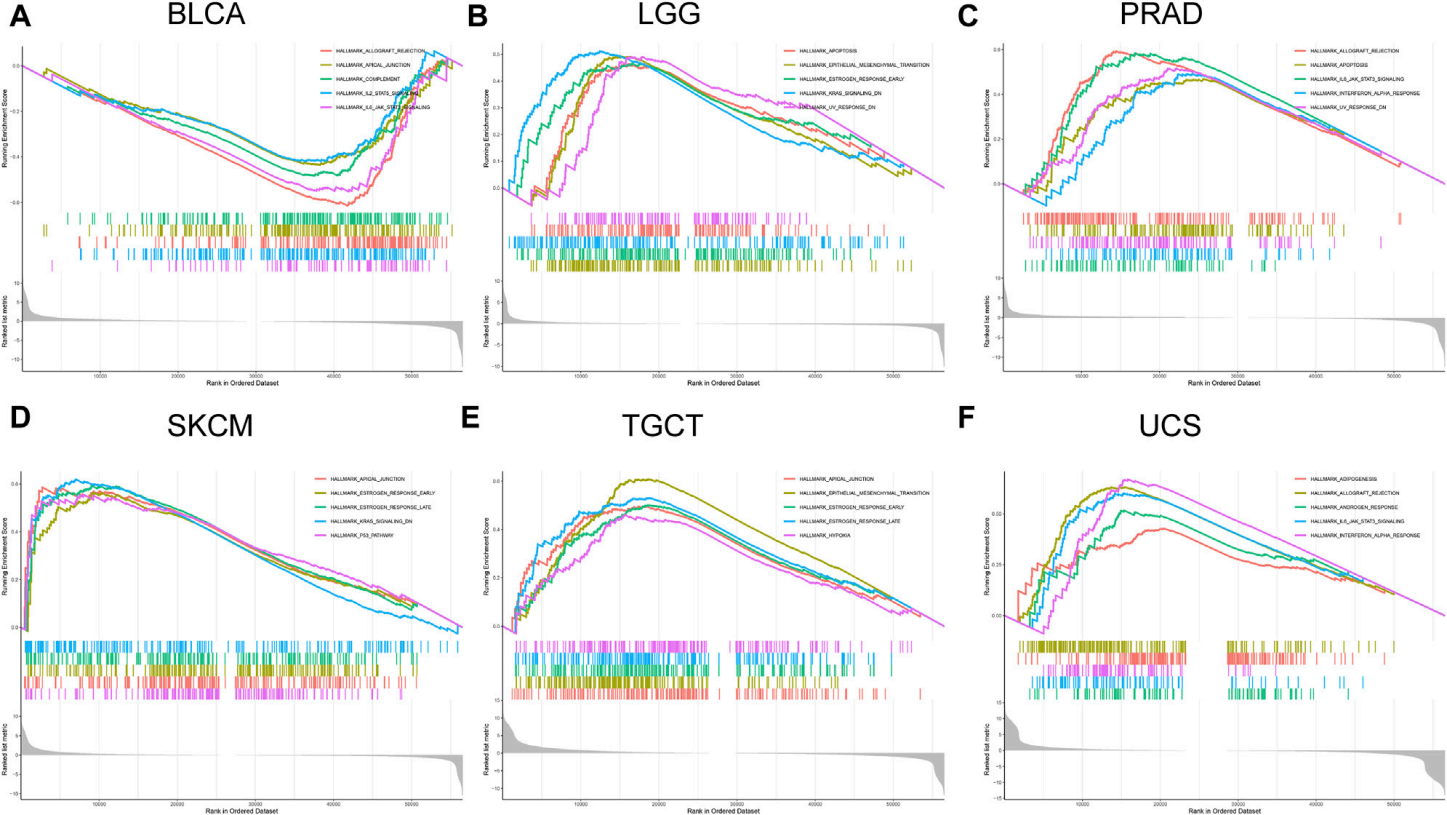
Association of forkhead box Q1 (FOXQ1) expression with cancer-related pathways in different cancers. Bladder urothelial carcinoma **(A)**, brain lower-grade glioma **(B)**, prostate adenocarcinoma **(C)**, skin cutaneous melanoma **(D)**, testicular germ cell tumors **(E)**, and uterine carcinosarcoma **(F)**.

The relationship between FOXQ1 expression and cancer-related pathway activation was examined to explore the mechanism underlying its regulation of tumor progression. The results showed that FOXQ1 was involved in the activation or repression of 33 pathways in 12 tumors (Table 1). For example, FOXQ1 expression inhibited activation of the IL6/JAK/STAT3 signaling pathway, IL2/STAT5 signaling pathway, allograft rejection pathway, apical junction pathway, and complement pathway in bladder urothelial carcinoma (Figure 7A). FOXQ1 expression participated in the activation of the epithelial–mesenchymal transition pathway, early estrogen response pathway, RAS signaling pathway, apoptosis pathway, and UV response pathway in brain lower-grade glioma (Figure 7B). FOXQ1 expression activated the following pathways in prostate adenocarcinoma: the IL6/JAK/STAT3 signaling pathway, interferon–alpha response pathway, UV response pathway, apoptosis pathway, and allograft rejection pathway (Figure 7C). FOXQ1 expression was additionally involved in activating the p53 pathway, apical junction pathway, early estrogen response pathway, late estrogen response pathway, and KRAS signaling pathway in skin cutaneous melanoma (Figure 7D). In testicular germ cell tumors, it participated in activation of the apical junction pathway, epithelial–mesenchymal transition pathway, early estrogen response pathway, late estrogen response pathway, and hypoxia pathway (Figure 7E). FOXQ1 also activated the following pathways in uterine carcinosarcoma: the androgen response pathway, IL6/JAK/STAT3 signaling pathway, interferon–alpha response pathway, adipogenesis pathway, and allograft rejection pathway (Figure 7F).

**TABLE 1:** Association of forkhead box Q1 expression with cancer-related pathway.

Cancer Type	Description	Enrichment score	*p* Value	Cancer Type	Description	Enrichment score	*p* Value
BLCA	IL6_JAK_STAT3_SIGNALING	−0.55	0.02	SKCM	APICAL_JUNCTION	0.59	0.01
BLCA	IL2_STAT5_SIGNALING	−0.42	0.02	SKCM	ESTROGEN_RESPONSE_EARLY	0.56	0.01
BLCA	ALLOGRAFT_REJECTION	−0.61	0.02	SKCM	ESTROGEN_RESPONSE_LATE	0.59	0.01
BLCA	APICAL_JUNCTION	−0.44	0.02	SKCM	KRAS_SIGNALING_DN	0.62	0.01
BLCA	COMPLEMENT	−0.48	0.02	SKCM	XENOBIOTIC_METABOLISM	0.56	0.01
BLCA	EPITHELIAL_MESENCHYMAL_TRANSITION	−0.60	0.02	SKCM	COAGULATION	0.62	0.02
BLCA	INFLAMMATORY_RESPONSE	−0.53	0.02	SKCM	DNA_REPAIR	−0.49	0.02
BLCA	INTERFERON_GAMMA_RESPONSE	−0.51	0.02	STAD	DNA_REPAIR	−0.53	0.03
BLCA	TNFA_SIGNALING_VIA_NFKB	−0.46	0.02	TGCT	APICAL_JUNCTION	0.50	0.01
BLCA	E2F_TARGETS	−0.40	0.04	TGCT	EPITHELIAL_MESENCHYMAL_TRANSITION	0.61	0.01
BLCA	G2M_CHECKPOINT	−0.40	0.04	TGCT	ESTROGEN_RESPONSE_EARLY	0.50	0.01
BLCA	KRAS_SIGNALING_DN	−0.40	0.04	TGCT	ESTROGEN_RESPONSE_LATE	0.53	0.01
BLCA	KRAS_SIGNALING_UP	−0.41	0.04	TGCT	HYPOXIA	0.46	0.01
CESC	INTERFERON_ALPHA_RESPONSE	0.50	0.05	TGCT	KRAS_SIGNALING_DN	0.56	0.01
CESC	DNA_REPAIR	−0.55	0.05	TGCT	KRAS_SIGNALING_UP	0.47	0.01
CHOL	PANCREAS_BETA_CELLS	0.78	0.02	TGCT	MYOGENESIS	0.57	0.01
CHOL	EPITHELIAL_MESENCHYMAL_TRANSITION	0.53	0.03	TGCT	XENOBIOTIC_METABOLISM	0.48	0.01
LGG	EPITHELIAL_MESENCHYMAL_TRANSITION	0.49	0.02	TGCT	COAGULATION	0.60	0.01
LGG	ESTROGEN_RESPONSE_EARLY	0.46	0.02	TGCT	PANCREAS_BETA_CELLS	0.77	0.02
LGG	KRAS_SIGNALING_DN	0.51	0.02	TGCT	GLYCOLYSIS	0.44	0.02
LGG	APOPTOSIS	0.46	0.03	TGCT	FATTY_ACID_METABOLISM	0.48	0.03
LGG	UV_RESPONSE_DN	0.49	0.05	TGCT	BILE_ACID_METABOLISM	0.54	0.03
PCPG	TNFA_SIGNALING_VIA_NFKB	0.55	0.02	TGCT	ANGIOGENESIS	0.65	0.03
PCPG	EPITHELIAL_MESENCHYMAL_TRANSITION	0.53	0.05	TGCT	UV_RESPONSE_DN	0.47	0.04
PRAD	IL6_JAK_STAT3_SIGNALING	0.58	0.03	THCA	INTERFERON_ALPHA_RESPONSE	0.65	0.04
PRAD	INTERFERON_ALPHA_RESPONSE	0.49	0.03	THCA	IL6_JAK_STAT3_SIGNALING	0.50	0.04
PRAD	UV_RESPONSE_DN	0.52	0.04	UCS	ANDROGEN_RESPONSE	0.52	0.03
PRAD	APOPTOSIS	0.47	0.04	UCS	IL6_JAK_STAT3_SIGNALING	0.60	0.03
PRAD	ALLOGRAFT_REJECTION	0.59	0.05	UCS	INTERFERON_ALPHA_RESPONSE	0.67	0.04
PRAD	APICAL_JUNCTION	0.45	0.05	UCS	ADIPOGENESIS	0.43	0.05
PRAD	COMPLEMENT	0.50	0.05	UCS	ALLOGRAFT_REJECTION	0.63	0.05
PRAD	EPITHELIAL_MESENCHYMAL_TRANSITION	0.52	0.05	UCS	COMPLEMENT	0.48	0.05
PRAD	ESTROGEN_RESPONSE_EARLY	0.49	0.05	UCS	EPITHELIAL_MESENCHYMAL_TRANSITION	0.49	0.05
PRAD	INFLAMMATORY_RESPONSE	0.59	0.05	UCS	ESTROGEN_RESPONSE_EARLY	0.48	0.05
PRAD	INTERFERON_GAMMA_RESPONSE	0.52	0.05	UCS	ESTROGEN_RESPONSE_LATE	0.52	0.05
PRAD	KRAS_SIGNALING_DN	0.49	0.05	UCS	FATTY_ACID_METABOLISM	0.49	0.05
PRAD	KRAS_SIGNALING_UP	0.53	0.05	UCS	IL2_STAT5_SIGNALING	0.52	0.05
PRAD	TNFA_SIGNALING_VIA_NFKB	0.54	0.05	UCS	INFLAMMATORY_RESPONSE	0.63	0.05
PRAD	IL2_STAT5_SIGNALING	0.48	0.05	UCS	INTERFERON_GAMMA_RESPONSE	0.64	0.05
PRAD	P53_PATHWAY	0.44	0.05	UCS	KRAS_SIGNALING_UP	0.52	0.05
SARC	OXIDATIVE_PHOSPHORYLATION	−0.42	0.04	UCS	TNFA_SIGNALING_VIA_NFKB	0.60	0.05
SKCM	P53_PATHWAY	0.56	0.01	UCS	XENOBIOTIC_METABOLISM	0.50	0.05

### Correlation of forkhead box Q1 expression with the tumor mutational burden and microsatellite instability in pan-cancer

**FIGURE 8:**
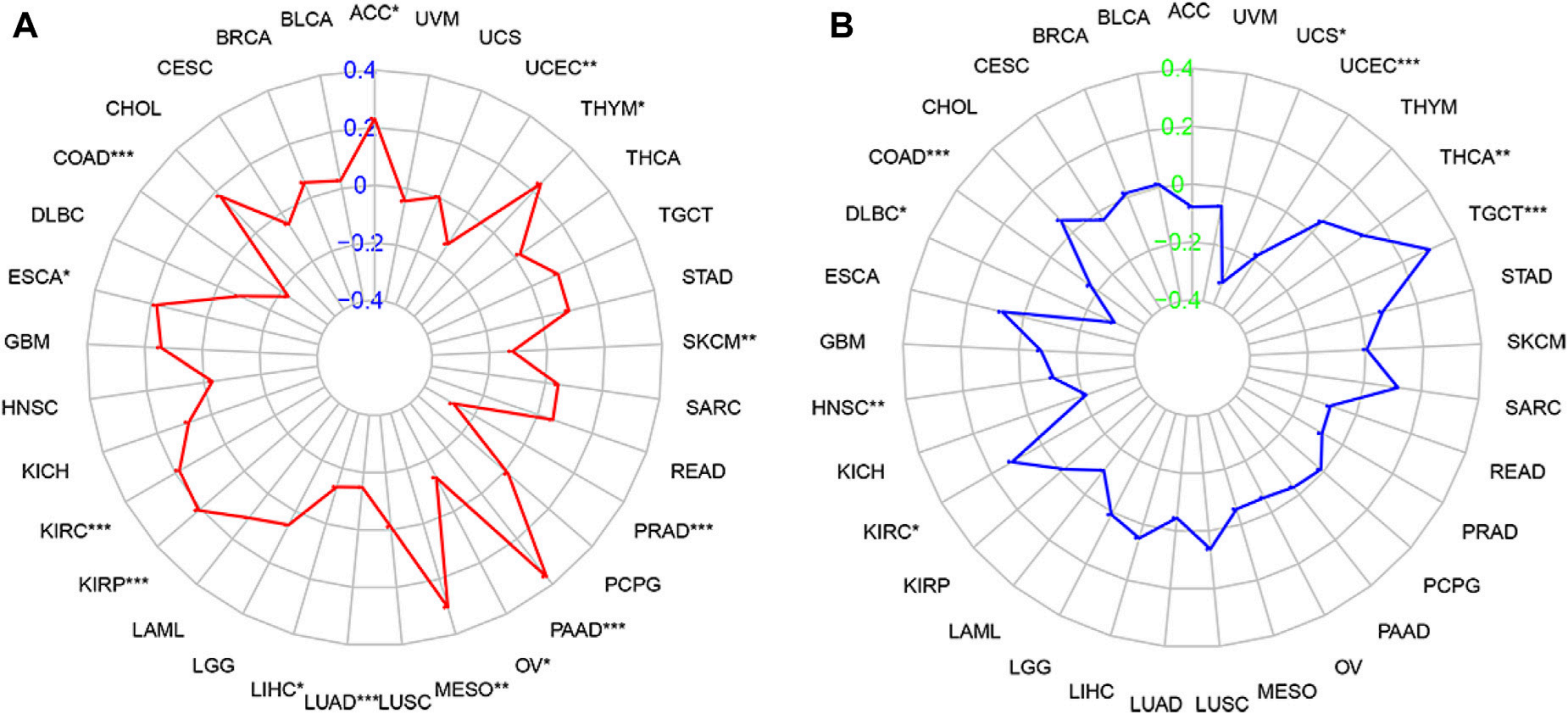
Correlation of forkhead box Q1 (FOXQ1) expression with tumor mutational burden (TMB) and microsatellite instability (MSI) in pan-cancer. **(A)** association of TMB with FOXQ1 expression in different cancers. The red curve represents the correlation coefficient, and the blue value represents the range. **(B)** relationship of MSI with FOXQ1 expression in different cancers. The blue curve represents the correlation coefficient, and the green value represents the range. *p < 0.05, **p < 0.01, and***p < 0.001.

We analyzed the relationship of FOXQ1 expression with the TMB and MSI in 33 types of tumors. Spearman correlation analysis showed that FOXQ1 expression was related to the TMB in 14 tumors, with FOXQ1 expression positively correlated with the TMB in cholangiocarcinoma, colon adenocarcinoma, esophageal carcinoma, kidney renal clear cell carcinoma, kidney renal papillary cell carcinoma, mesothelioma, pancreatic adenocarcinoma, and thyroid carcinoma and negatively correlated with the TMB in liver hepatocellular carcinoma, lung adenocarcinoma, ovarian serous cystadenocarcinoma, prostate adenocarcinoma, skin cutaneous melanoma and uterine corpus endometrial carcinoma (Figure 8A).

In addition, FOXQ1 expression was related to MSI in 8 types of tumors. FOXQ1 expression was negatively correlated with MSI in colon adenocarcinoma, lymphoid neoplasm diffuse large B-cell lymphoma, head and neck squamous cell carcinoma, uterine corpus endometrial carcinoma, and uterine carcinosarcoma and positively correlated with MSI in kidney renal clear cell carcinoma, thyroid carcinoma, and testicular germ cell tumors (Figure 8B).

### Correlation between forkhead box Q1 expression and the tumor microenvironment in pan-cancer

**FIGURE 9:**
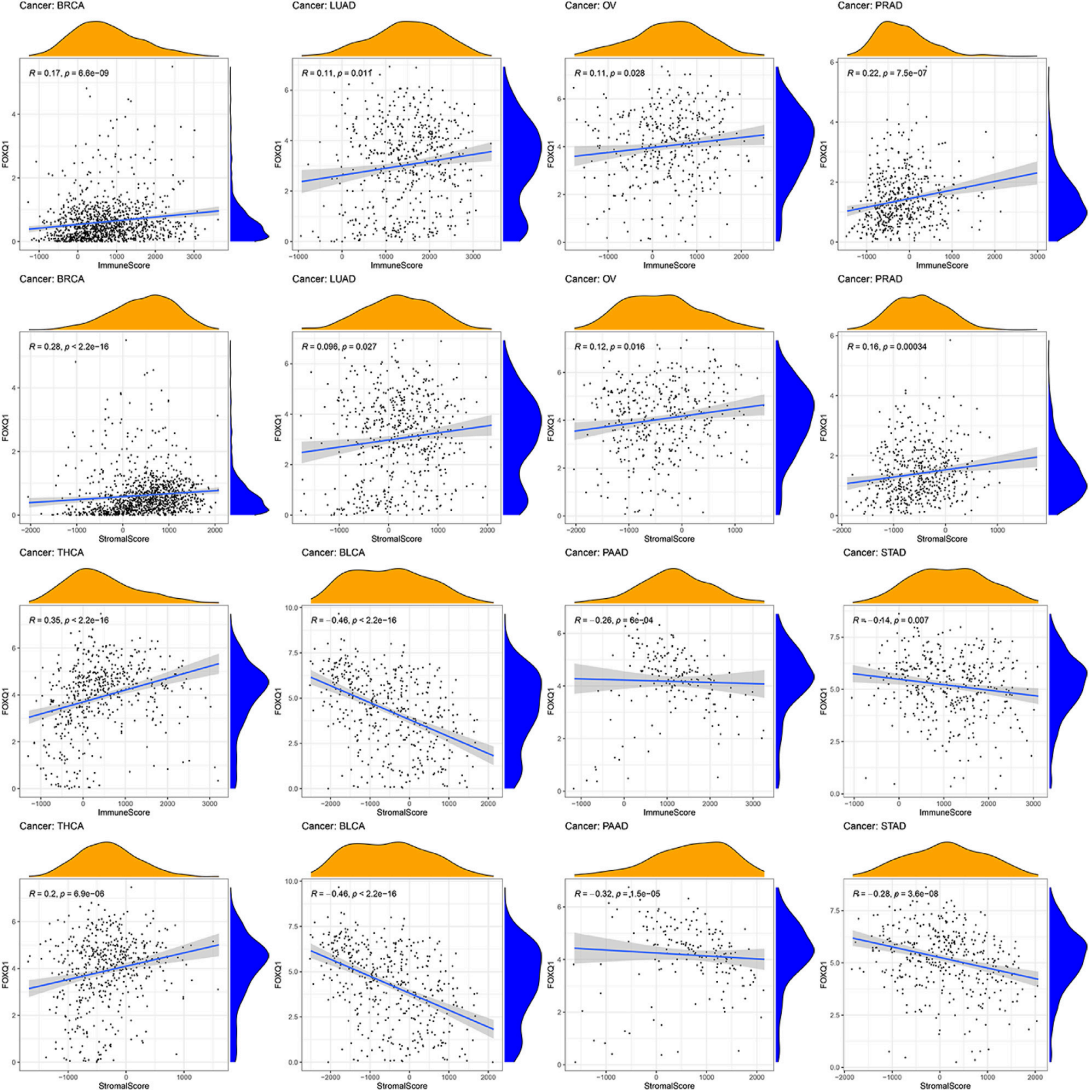
Relationship of forkhead box Q1 (FOXQ1) expression with tumor microenvironment across different cancer types. FOXQ1 expression was positively correlated with stromal score and immune score in breast cancer, prostate adenocarcinoma, thyroid carcinoma, lung adenocarcinoma, and ovarian serous cystadenocarcinoma and was negatively correlated with bladder urothelial carcinoma, pancreatic adenocarcinoma, and stomach adenocarcinoma.

The ESTIMATE algorithm was adopted to evaluate the association of FOXQ1 expression with the TME. The results proved that FOXQ1 expression was positively correlated with the stromal and immune score in breast invasive carcinoma, prostate adenocarcinoma, thyroid carcinoma, lung adenocarcinoma, and ovarian serous cystadenocarcinoma. In other words, with an increase in stromal and immune cells, FOXQ1 expression was increased. By contrast, its expression was negatively correlated with the stromal and immune score in pancreatic adenocarcinoma, bladder urothelial carcinoma, and stomach adenocarcinoma, which suggested that with an increase in the stromal and immune score, FOXQ1 expression was decreased (Figure 9).

### Association of forkhead box Q1 expression with immune cell infiltration in pan-cancer

**FIGURE 10:**
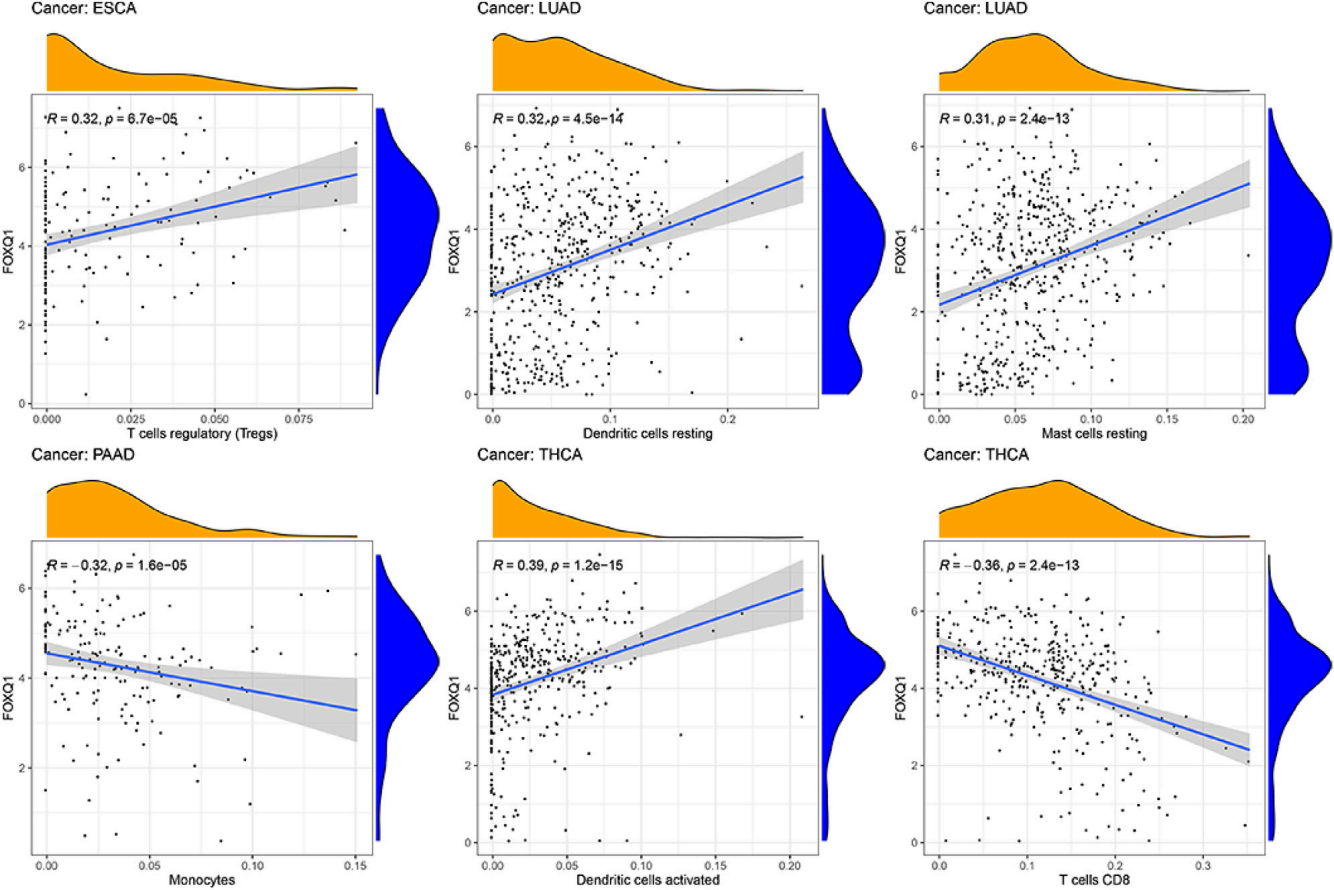
Correlation of forkhead box Q1 expression with immune cell infiltration in esophageal carcinoma, lung adenocarcinoma, pancreatic adenocarcinoma, and thyroid carcinoma.

The influence of FOXQ1 expression on the infiltration of 22 types of immune cells was assessed with the CIBERSORT algorithm. We found that FOXQ1 expression was related to the infiltration of the 22 different immune cell types in different cancers, such as breast invasive carcinoma (*n* = 12), thyroid carcinoma (*n* = 11), bladder urothelial carcinoma (*n* = 10), and lung adenocarcinoma (*n* = 10) (Table 2); other results are shown in Supplementary Table S3. For instance, FOXQ1 expression was positively correlated with T regulatory cells (T regs) in esophageal carcinoma (Figure 10). Its expression was also positively correlated with resting dendritic cells and resting mast cells in lung adenocarcinoma and was negatively correlated with monocytes in pancreatic adenocarcinoma (Figure 10). In addition, the TIMER heatmap is shown in Supplementary Figure S1.

**TABLE 2:** Association of forkhead box Q1 expression with immune cell infiltration.

Cancer type	Immune cell types	Cor	*p* Value	Cancer type	Immune cell types	Cor	*p* Value
BRCA	Macrophages M2	−0.23	<0.001	THCA	T cells CD8	−0.36	<0.001
BRCA	Dendritic cells activated	0.19	<0.001	THCA	Dendritic cells activated	0.39	<0.001
BRCA	Dendritic cells resting	0.13	<0.001	THCA	T cells regulatory (T regs)	0.27	<0.001
BRCA	B cells naive	0.11	<0.001	THCA	Dendritic cells resting	0.24	<0.001
BRCA	T cells regulatory (T regs)	0.10	<0.001	THCA	B cells naive	0.16	<0.001
BRCA	T cells CD4 memory resting	0.10	<0.001	THCA	Macrophages M0	0.16	<0.001
BRCA	Macrophages M1	0.09	<0.001	THCA	T cells CD4 memory resting	0.12	0.02
BRCA	Plasma cells	0.07	0.02	THCA	NK cells resting	−0.11	0.03
BRCA	T cells CD4 memory activated	0.07	0.03	THCA	Macrophages M1	−0.11	0.03
BRCA	Monocytes	−0.06	0.04	THCA	Macrophages M2	−0.12	0.02
BRCA	T cells gamma delta	−0.08	0.01	THCA	B cells memory	−0.22	<0.001
BRCA	Mast cells resting	−0.12	<0.001	LUAD	Macrophages M0	−0.24	<0.001
BLCA	Macrophages M2	−0.23	<0.001	LUAD	Dendritic cells resting	0.32	<0.001
BLCA	T cells regulatory (T regs)	0.23	<0.001	LUAD	Mast cells resting	0.31	<0.001
BLCA	B cells memory	0.16	<0.001	LUAD	Dendritic cells activated	0.17	<0.001
BLCA	Dendritic cells activated	0.13	0.02	LUAD	T cells regulatory (T regs)	0.13	<0.001
BLCA	T cells follicular helper	0.13	0.02	LUAD	T cells CD4 memory resting	0.12	0.01
BLCA	Dendritic cells resting	0.11	0.05	LUAD	Monocytes	0.09	0.05
BLCA	NK cells activated	−0.17	<0.001	LUAD	T cells CD4 memory activated	−0.15	<0.001
BLCA	Monocytes	−0.19	<0.001	LUAD	Plasma cells	−0.16	<0.001
BLCA	Neutrophils	−0.19	<0.001	LUAD	Macrophages M1	−0.18	<0.001
BLCA	Macrophages M0	−0.20	<0.001				

### Coexpression of immune-related genes with forkhead box Q1 in pan-cancer

**FIGURE 11:**
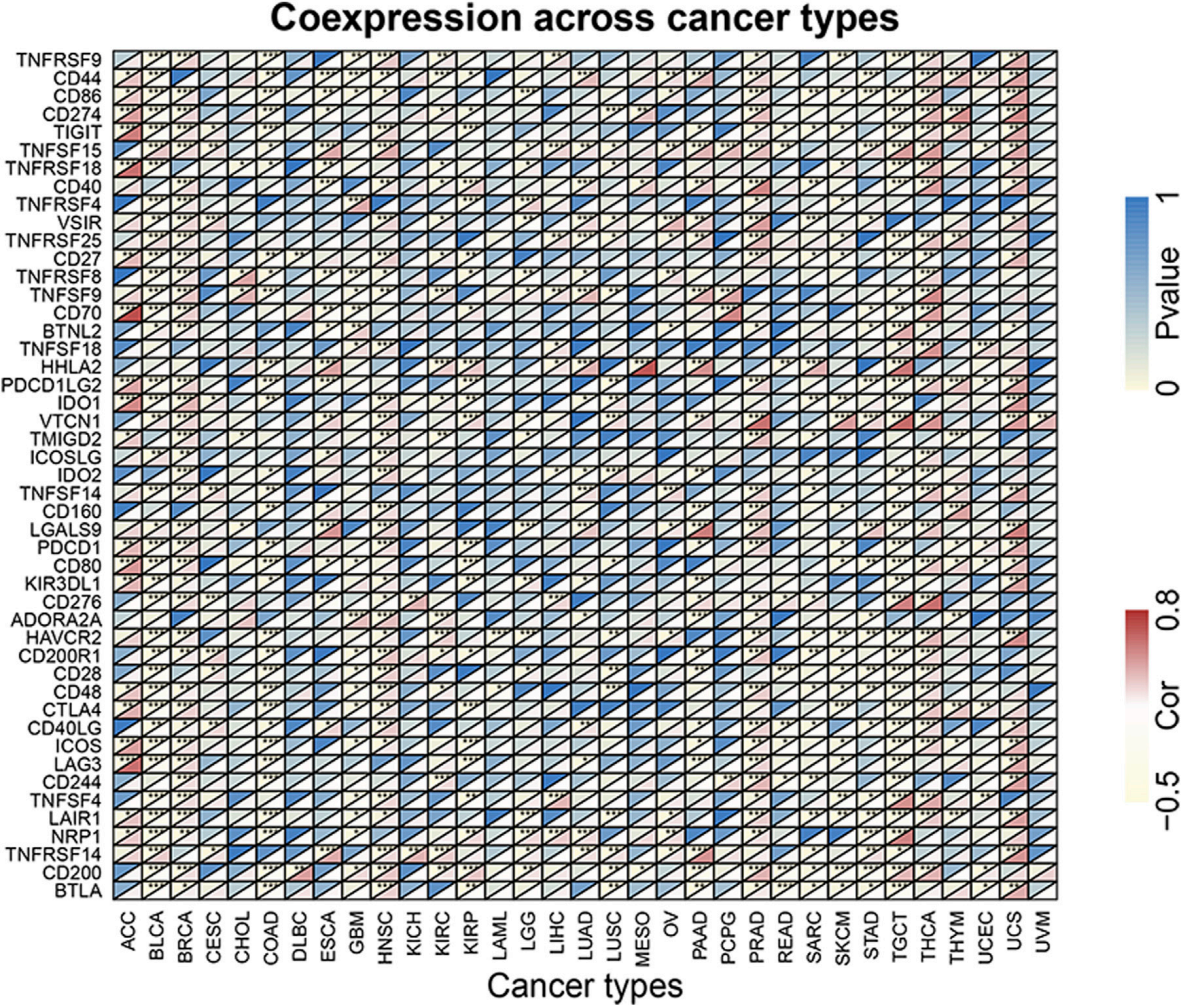
Coexpression of forkhead box Q1 with immune-related genes in pan-cancer. The red color represents the correlation coefficient, and the blue color represents the *p* value. *p < 0.05, **p < 0.01, and ***p < 0.001.

To explore the correlation of FOXQ1 expression with immune-related genes in pan-cancer, coexpression analysis was performed. This work revealed that FOXQ1 was coexpressed with 47 immune-related genes in different cancers (*p* < 0.05), such as CD44, CD86, CD274, TNFRSF9, TIGIT, TNFSF15, TNFRSF18, TNFRSF4, VSIR, and TNFRSF25 (Figure 11). The findings also indicated that the coexpression correlation of FOXQ1 with immune-related genes was mainly focused on adrenocortical carcinoma, breast invasive carcinoma, head and neck squamous cell carcinoma, prostate adenocarcinoma, thyroid carcinoma, testicular germ cell tumors, uterine carcinosarcoma, and thymoma (Figure 11 and Supplementary Figure S2).

## Discussion

This joint analysis of FOXQ1 at different levels is the first to be conducted using pan-cancer expression, survival, and mutation data from TCGA, Oncomine, and CCLE databases. Our results comprehensively summarize the FOXQ1 expression profile at tissue and cell levels and the association of its expression with prognosis, clinicopathological characteristics, cancer-related pathways, TMB, MSI, TME, immune cell infiltration, and immune-related genes. These results deepen our understanding of the FOXQ1 profile in pan-cancer, provide important clues for building immune therapy regimens targeting FOXQ1, and significantly help to guide the exploration of FOXQ1 pathogenic mechanisms in pan-cancer.

At the tissue level, TCGA and Oncomine databases showed that FOXQ1 was upregulated in colon adenocarcinoma, lung adenocarcinoma, lung squamous cell carcinoma, thyroid carcinoma, and liver hepatocellular carcinoma and downregulated in kidney cancer and prostate adenocarcinoma. At the cell level, the CCLE database suggested that FOXQ1 was expressed in 1,057 cell lines from 36 tumors, with the background expression level high in pancreatic adenocarcinoma, liver hepatocellular carcinoma, colon adenocarcinoma, lung cancer, and thyroid carcinoma. FOXQ1 has been reported to be highly significantly expressed in colon adenocarcinoma and rectum adenocarcinoma (Kaneda et al., 2010). FOXQ1 is also upregulated in lung cancer and thyroid carcinoma and has been related to poor prognosis (Feng et al., 2012; Li et al., 2019). Our study results indicate that FOXQ1 was upregulated in colon adenocarcinoma, liver hepatocellular carcinoma, pancreatic adenocarcinoma, and ovarian serous cystadenocarcinoma and downregulated in kidney cancer tissue and cell lines, which hints at a major role for FOXQ1 in tumor occurrence and development and its potential as a tumor diagnostic biomarker.

We also analyzed the correlation of FOXQ1 expression with prognostic and clinicopathological parameters in 33 tumor types. Kaplan–Meier and Cox regression analyses indicated that FOXQ1 expression decreased the OS of skin cutaneous melanoma and thymoma patients. Moreover, its expression influenced the DSS of prostate adenocarcinoma and liver hepatocellular carcinoma. FOXQ1 expression was also associated with poor DFS in pancreatic adenocarcinoma. In addition, FOXQ1 expression affected the tumor progression of bladder urothelial carcinoma, skin cutaneous melanoma, colon adenocarcinoma, thyroid carcinoma, and testicular germ cell tumors and regulated the differentiation degree of tumor cells in bladder urothelial carcinoma, stomach adenocarcinoma, uterine corpus endometrial carcinoma, brain lower-grade glioma, and liver hepatocellular carcinoma. A meta-analysis determined that FOXQ1 expression was related to poor prognosis in malignant solid tumors (Cui et al., 2017). FOXQ1 expression also regulates the progression of different tumor types (Gao et al., 2012; Zhang et al., 2015). It is well known that different factors can affect tumor prognosis and that the interaction among factors coregulates the homeostasis of tumor patients. Patient prognosis is also affected when homeostasis is disturbed by abnormal factors and becomes unbalanced. This study is the first to find that FOXQ1 can exert distinct influences on various tumors may be an abnormal factor causing dyshomeostasis and has the potential to be a prognostic biomarker for tumors. These findings also hinted that the pathogenic mechanism of FOXQ1 differed among tumors of different types.

The correlation of FOXQ1 expression with cancer-related pathway activity was explored to determine the potential mechanism of FOXQ1 in pan-cancer. The results indicated that FOXQ1 expression was involved in the activation and inactivation of 33 pathways in 12 types of tumors, including the IL6/JAK/STAT3 signaling pathway, allograft rejection pathway, apical junction pathway, epithelial–mesenchymal transition pathway, early estrogen response pathway, interferon–alpha response pathway, RAS signaling pathway, and apoptosis pathway. It has been reported that an activity change in the IL6/JAK/STAT3 signaling pathway can regulate liver hepatocellular carcinoma, chronic myeloid leukemia, and glioma (Yao et al., 2016; Yang et al., 2018; Ni et al., 2020). The epithelial–mesenchymal transition pathway enhances the proliferation, invasion, and migration of tumor cells (Mittal, 2018); influences the function and number of immune cells in the TME; and regulates anticancer immunity (Dongre and Weinberg, 2019). The estrogen response pathway also regulates the TME and is a potential tumor endocrine therapy target (Yamaguchi, 2007; Rothenberger et al., 2018). The interferon–alpha response pathway induces cell tumor cell apoptosis and participates in anticancer immunity (Brassard et al., 2002; Shi et al., 2016). We showed that FOXQ1 expression could cause activity changes in various cancer-related pathways in 12 different tumor types, such as bladder urothelial carcinoma, prostate adenocarcinoma, skin cutaneous melanoma, and brain lower-grade glioma. Therefore, we predicted that FOXQ1 was able to regulate tumor progression via these pathways. These conclusions provide important clues for pathogenic mechanistic research for FOXQ1.

Spearman correlation analysis was used to identify the relationship of FOXQ1 expression with the TMB and MSI. The results indicated that FOXQ1 expression was associated with the TMB in 14 tumor types, such as cholangiocarcinoma, colon adenocarcinoma, and esophageal carcinoma, and with MSI in 8 tumor types, such as head and neck squamous cell carcinoma, kidney renal clear cell carcinoma, and thyroid carcinoma. A significant correlation has been found between the TMB with the tumor immunotherapy response and the objective response rate of tumor patients (Yarchoan et al., 2017; Ritterhouse, 2019). The PFS of high TMB patients is significantly improved in nonsmall cell lung cancer patients treated with nivolumab plus ipilimumab, which suggests that the TMB could act as an independent biomarker (Hellmann et al., 2018). It has been reported that MSI could also be an immune response biomarker for tumor patients (Schrock et al., 2019). The TMB could be regarded as a stratified biomarker for the immune response in MSI colon adenocarcinoma patients (Schrock et al., 2019). We initially found a significant correlation of FOXQ1 expression with the TMB and MSI in different tumors, and we predicted that it has the potential to be a tumor immunotherapy target.

The signal transduction pathways involved in FOXQ1 expression might regulate the immune response, which explains the association of FOXQ1 expression with the TME, immune cell infiltration, and immune-related genes. The results showed that with an increase in stromal and immune cells, FOXQ1 expression was increased in breast invasive carcinoma, pancreatic adenocarcinoma, thyroid carcinoma, lung adenocarcinoma, and ovarian serous cystadenocarcinoma, whereas its expression was decreased in pancreatic adenocarcinoma, bladder urothelial carcinoma, and stomach adenocarcinoma. FOXQ1 expression was closely related to the infiltration of 22 immune cell types in 31 tumors, including resting memory CD4 T cells, T follicular helper cells, memory B cells, activated NK cells, plasma cells, naive B cells, M0 macrophages, T regs, resting dendritic cells, and resting mast cells. FOXQ1 was coexpressed with 47 immune-related genes in different cancers, such as CD44, CD86, CD274, TNFRSF9, TIGIT, TNFSF15, TNFRSF18, TNFRSF4, VSIR, and TNFRSF25. The role of FOXQ1 in immunology has been preliminarily confirmed, with previous research showing that cancer-related macrophages induce epithelial–mesenchymal transition by regulating the JAK2/STAT3/miR-506-3p/FOXQ1 axis, thereby enhancing the invasion and migration of colorectal cancer cells (Wei et al., 2019). FOXQ1 may influence the biological process by regulating IL-6 and IL-8 (Wang et al., 2017). Our study is the first to explore the association of FOXQ1 expression with tumor immunity from different perspectives. Our results show that FOXQ1 may play a role in mediating tumor immunity, which has major usefulness in guiding the exploitation of new anticancer immune therapeutic targets.

In conclusion, this study is the first to prove that FOXQ1 expression is closely associated with prognosis, clinicopathological parameters, cancer-related pathways, TMB, MSI, TME, immune cell infiltration, and immune-related genes via TCGA, Oncomine, and CCLE databases. We have explored in depth the expression characteristics and possible pathogenic mechanisms of FOXQ1 in tumors, which provide new ideas for the development of novel immunotherapeutic targets.

## Data Availability

The original contributions presented in the study are included in the article/Supplementary Material, further inquiries can be directed to the corresponding authors.

## Glossary

ACC: Adrenocortical carcinoma
BLCA: Bladder Urothelial Carcinoma
BRCA: Breast invasive carcinoma
CCLE: Cancer Cell Line Encyclopedia
CESC: Cervical squamous cell carcinoma and endocervical adenocarcinoma
CHOL: Cholangiocarcinoma
COAD: Colon adenocarcinoma
DLBC: Lymphoid neoplasm diffuse large B-cell lymphoma
ESCA: Esophageal carcinoma
FOXQ1: Forkhead box Q1
GBM: Glioblastoma multiforme
HNSC: Head and neck squamous cell carcinoma
KICH: Kidney chromophobe
KIRC: Kidney renal clear cell carcinoma
KIRP: Kidney renal papillary cell carcinoma
LAML: Acute myeloid leukemia
LGG: Brain lower-grade glioma
LIHC: Liver hepatocellular carcinoma
LUAD: Lung adenocarcinoma
LUSC: Lung squamous cell carcinoma
MESO: Mesothelioma
MSI: Microsatellite instability
OV: Ovarian serous cystadenocarcinoma
PAAD: Pancreatic adenocarcinoma
PCPG: Pheochromocytoma and paraganglioma
PRAD: Prostate adenocarcinoma
READ: Rectum adenocarcinoma
SARC: Sarcoma
SKCM: Skin cutaneous melanoma
STAD: Stomach adenocarcinoma
TCGA: The Cancer Genome Atlas
TGCT: Testicular germ cell tumors
THCA: Thyroid carcinoma
THYM: Thymoma
TMB: Tumor mutational burden
TME: Tumor microenvironment
UCEC: Uterine corpus endometrial carcinoma
UCS: Uterine carcinosarcoma
UVM: Uveal melanoma
