# Supplementary material for: Pan-cancer analysis of forkhead box Q1 as a potential prognostic and immunological biomarker
Source: Front Genet. 2022 Sep 1;13:944970. doi: 10.3389/fgene.2022.944970 (PMC9475120; doi:10.3389/fgene.2022.944970)
Supplement: Supplementary file 1 [file DataSheet1.docx]

**SUPPLEMENTARY MATERIAL**

| **Pan-Cancer Analysis of FOXQ1 as a Potential Prognostic and Immunological Biomarker**  **TABLE S1 The sample size of prognosis analysis in pan-cancer.** | | | | |
| --- | --- | --- | --- | --- |
| **CancerType** | **OS** | **DSS** | **DFS** | **PFS** |
| ACC | 92 | 90 | 53 | 92 |
| BLCA | 436 | 418 | 195 | 436 |
| BRCA | 1236 | 1206 | 1055 | 1236 |
| CESC | 312 | 308 | 178 | 312 |
| CHOL | 45 | 43 | 32 | 45 |
| COAD | 545 | 529 | 221 | 545 |
| DLBC | 48 | 48 | 28 | 48 |
| ESCA | 204 | 200 | 99 | 204 |
| GBM | 602 | 561 | 3 | 602 |
| HNSC | 604 | 574 | 143 | 604 |
| KICH | 91 | 91 | 42 | 91 |
| KIRC | 944 | 923 | 165 | 944 |
| KIRP | 325 | 348 | 197 | 352 |
| LAML | 200 | 67 | 0 | 0 |
| LGG | 529 | 521 | 137 | 529 |
| LIHC | 438 | 425 | 372 | 438 |
| LUAD | 641 | 601 | 384 | 641 |
| LUSC | 623 | 549 | 365 | 623 |
| MESO | 87 | 66 | 15 | 87 |
| OV | 602 | 564 | 299 | 604 |
| PAAD | 196 | 188 | 71 | 196 |
| PCPG | 187 | 187 | 164 | 187 |
| PRAD | 566 | 564 | 396 | 396 |
| READ | 183 | 177 | 51 | 183 |
| SKCM | 472 | 466 | 0 | 472 |
| SARC | 271 | 265 | 160 | 271 |
| STAD | 511 | 470 | 285 | 511 |
| TGCT | 139 | 139 | 110 | 139 |
| THCA | 580 | 574 | 408 | 580 |
| THYM | 126 | 126 | 0 | 126 |
| UCEC | 583 | 581 | 452 | 583 |
| UCS | 57 | 55 | 27 | 57 |
| UVM | 80 | 80 | 0 | 80 |

OS, Overall Survival; DSS, Disease-Specific Survival; DFS, Disease-Free Survival; PFS, Progression-Free Survival.

| **TABLE S2. Basic information of 33 type of cancers from TCGA** | | |
| --- | --- | --- |
| **Cancer Type** | **Cancer Cases** | **Normal Cases** |
| Kidney renal clear cell carcinoma | 535 | 72 |
| Kidney renal papillary cell carcinoma | 289 | 32 |
| Kidney Chromophobe | 65 | 24 |
| Colon adenocarcinoma | 471 | 41 |
| Lung squamous cell carcinoma | 501 | 49 |
| Lung adenocarcinoma | 526 | 59 |
| Uterine Corpus Endometrial Carcinoma | 548 | 35 |
| Stomach adenocarcinoma | 375 | 32 |
| Prostate adenocarcinoma | 499 | 52 |
| Bladder Urothelial Carcinoma | 411 | 19 |
| Breast invasive carcinoma | 1104 | 113 |
| Cervical squamous cell carcinoma and endocervical adenocarcinoma | 306 | 3 |
| Cholangiocarcinoma | 36 | 9 |
| Esophageal carcinoma | 162 | 11 |
| Glioblastoma multiforme | 168 | 5 |
| Head and Neck squamous cell carcinoma | 502 | 44 |
| Liver hepatocellular carcinoma | 374 | 50 |
| Pancreatic adenocarcinoma | 178 | 4 |
| Pheochromocytoma, and Paraganglioma | 183 | 3 |
| Rectum adenocarcinoma | 167 | 10 |
| Sarcoma | 263 | 2 |
| Skin Cutaneous Melanoma | 471 | 1 |
| Thyroid carcinoma | 510 | 58 |
| Thymoma | 119 | 2 |
| Uterine Carcinosarcoma | 56 | 0 |
| Uveal Melanoma | 80 | 0 |
| Adrenocortical carcinoma | 79 | 0 |
| Lymphoid Neoplasm Diffuse Large B-cell Lymphoma | 48 | 0 |
| Testicular Germ Cell Tumors | 156 | 0 |
| Ovarian serous cystadenocarcinoma | 379 | 0 |
| Acute Myeloid Leukemia | 151 | 0 |
| Brain Lower Grade Glioma | 529 | 0 |
| Mesothelioma | 86 | 0 |
| TCGA, The Cancer Genome Atlas | | |

| **TABLE S3 Association of FOXQ1 expression with immune cell infiltration.** | | | |
| --- | --- | --- | --- |
| **Cancertype** | **immune cell types** | **Cor** | ***P* value** |
| ACC | T cells follicular helper | 0.36 | 0.02 |
| ACC | T cells CD4 memory resting | -0.39 | 0.01 |
| ACC | Mast cells resting | -0.40 | 0.01 |
| CESC | Macrophages M2 | -0.21 | <0.001 |
| CESC | Macrophages M0 | -0.22 | <0.001 |
| CHOL | Plasma cells | 0.44 | 0.01 |
| CHOL | NK cells activated | -0.42 | 0.02 |
| CHOL | B cells memory | -0.50 | <0.001 |
| COAD | Macrophages M0 | 0.17 | <0.001 |
| DLBC | Macrophages M0 | 0.33 | 0.02 |
| DLBC | B cells naive | -0.31 | 0.03 |
| ESCA | T cells regulatory (T regs) | 0.32 | <0.001 |
| ESCA | B cells naive | 0.28 | <0.001 |
| ESCA | T cells CD4 memory resting | 0.16 | 0.05 |
| ESCA | Macrophages M0 | -0.18 | 0.03 |
| ESCA | Macrophages M2 | -0.18 | 0.03 |
| ESCA | Macrophages M1 | -0.22 | 0.01 |
| ESCA | Dendritic cells resting | -0.25 | <0.001 |
| GBM | Macrophages M2 | 0.22 | <0.001 |
| GBM | B cells memory | -0.16 | 0.04 |
| GBM | Plasma cells | -0.19 | 0.01 |
| GBM | T cells gamma delta | -0.20 | 0.01 |
| HNSC | B cells naive | 0.15 | <0.001 |
| HNSC | Plasma cells | 0.12 | 0.01 |
| HNSC | Mast cells resting | 0.11 | 0.01 |
| HNSC | T cells CD4 memory resting | 0.10 | 0.03 |
| HNSC | Macrophages M0 | -0.10 | 0.03 |
| HNSC | Macrophages M2 | -0.16 | <0.001 |
| HNSC | Mast cells activated | -0.17 | <0.001 |
| HNSC | Neutrophils | -0.17 | <0.001 |
| KICH | Macrophages M0 | 0.35 | 0.04 |
| KIRC | T cells follicular helper | 0.14 | <0.001 |
| KIRC | T cells CD8 | 0.11 | 0.01 |
| KIRC | Monocytes | 0.09 | 0.03 |
| KIRC | NK cells activated | 0.09 | 0.04 |
| KIRC | Dendritic cells resting | 0.09 | 0.05 |
| KIRC | Macrophages M0 | -0.16 | <0.001 |
| KIRP | Macrophages M2 | 0.17 | 0.01 |
| KIRP | Macrophages M0 | 0.13 | 0.04 |
| KIRP | Plasma cells | -0.16 | 0.01 |
| KIRP | T cells CD8 | -0.16 | 0.01 |
| KIRP | T cells CD4 memory activated | -0.23 | <0.001 |
| LAML | T cells CD4 memory activated | 0.20 | 0.01 |
| LGG | Macrophages M1 | 0.27 | <0.001 |
| LGG | B cells naive | 0.14 | 0.01 |
| LGG | T cells follicular helper | 0.13 | 0.02 |
| LGG | Macrophages M2 | -0.12 | 0.02 |
| LGG | Monocytes | -0.21 | <0.001 |
| LIHC | Macrophages M0 | 0.15 | 0.01 |
| LIHC | T cells follicular helper | 0.14 | 0.02 |
| LIHC | T cells regulatory (T regs) | 0.14 | 0.02 |
| LIHC | Monocytes | -0.11 | 0.05 |
| LIHC | NK cells activated | -0.12 | 0.03 |
| LIHC | T cells CD8 | -0.14 | 0.01 |
| LIHC | Macrophages M2 | -0.14 | 0.02 |
| LUSC | Dendritic cells activated | 0.20 | <0.001 |
| LUSC | Eosinophils | -0.09 | 0.04 |
| LUSC | Macrophages M1 | -0.10 | 0.02 |
| LUSC | Macrophages M2 | -0.18 | <0.001 |
| MESO | Monocytes | -0.25 | 0.02 |
| OV | Monocytes | 0.25 | <0.001 |
| OV | T cells CD4 memory resting | 0.25 | <0.001 |
| OV | Mast cells activated | 0.12 | 0.03 |
| OV | T cells follicular helper | -0.14 | 0.01 |
| OV | T cells CD4 memory activated | -0.17 | <0.001 |
| PAAD | Macrophages M0 | 0.28 | <0.001 |
| PAAD | B cells memory | 0.23 | <0.001 |
| PAAD | T cells CD4 memory resting | 0.19 | 0.02 |
| PAAD | Dendritic cells activated | 0.17 | 0.03 |
| PAAD | B cells naive | -0.17 | 0.03 |
| PAAD | T cells CD8 | -0.24 | <0.001 |
| PAAD | T cells CD4 memory activated | -0.27 | <0.001 |
| PAAD | Monocytes | -0.32 | <0.001 |
| PCPG | Macrophages M2 | -0.24 | 0.05 |
| PCPG | T cells regulatory (T regs) | -0.31 | 0.01 |
| PRAD | Dendritic cells resting | 0.28 | <0.001 |
| PRAD | B cells memory | -0.11 | 0.04 |
| PRAD | Macrophages M2 | -0.20 | <0.001 |
| READ | Neutrophils | 0.18 | 0.03 |
| READ | T cells CD4 memory resting | -0.24 | <0.001 |
| SKCM | Neutrophils | 0.23 | <0.001 |
| SKCM | Dendritic cells activated | 0.13 | 0.01 |
| SKCM | Mast cells resting | 0.11 | 0.02 |
| SKCM | Dendritic cells resting | 0.10 | 0.04 |
| SKCM | Plasma cells | -0.10 | 0.05 |
| SKCM | Monocytes | -0.10 | 0.04 |
| SKCM | B cells naive | -0.10 | 0.04 |
| SKCM | T cells CD4 memory activated | -0.13 | 0.01 |
| STAD | Plasma cells | 0.15 | <0.001 |
| STAD | NK cells activated | 0.14 | 0.01 |
| STAD | Macrophages M1 | 0.10 | 0.04 |
| STAD | Neutrophils | -0.12 | 0.02 |
| STAD | Macrophages M2 | -0.15 | <0.001 |
| STAD | Eosinophils | -0.25 | <0.001 |
| TGCT | Macrophages M2 | 0.59 | <0.001 |
| TGCT | Dendritic cells activated | 0.26 | <0.001 |
| TGCT | Mast cells resting | 0.21 | 0.01 |
| TGCT | B cells memory | 0.19 | 0.02 |
| TGCT | Macrophages M1 | -0.19 | 0.02 |
| TGCT | Plasma cells | -0.32 | <0.001 |
| TGCT | T cells CD4 memory activated | -0.42 | <0.001 |
| TGCT | B cells naive | -0.50 | <0.001 |
| THYM | T cells gamma delta | 0.31 | <0.001 |
| THYM | Macrophages M0 | 0.27 | <0.001 |
| THYM | Macrophages M1 | 0.24 | 0.01 |
| THYM | Dendritic cells activated | 0.18 | 0.05 |
| THYM | Dendritic cells resting | -0.23 | 0.01 |
| THYM | NK cells resting | -0.27 | <0.001 |
| UCEC | B cells naive | 0.10 | 0.03 |
| UCEC | B cells memory | -0.10 | 0.02 |
| UCEC | Macrophages M1 | -0.11 | 0.02 |
| UCEC | Macrophages M2 | -0.16 | <0.001 |
| UCS | Dendritic cells resting | 0.36 | 0.02 |


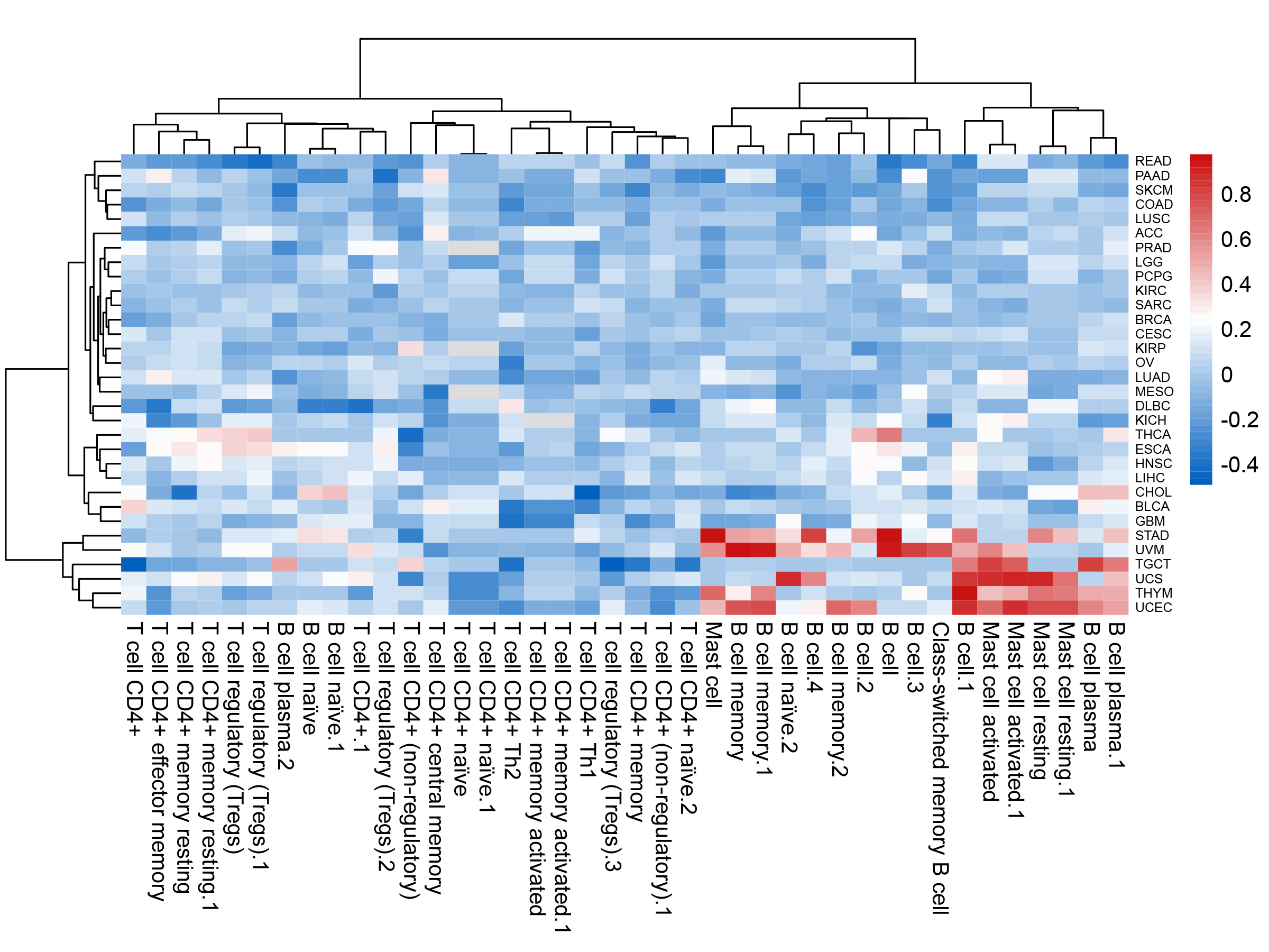


FIGURE S1: The heatmap of FOXQ1 expression with T cell CD4^+^, Tregs, B cell and mast cell in pan-cancer by TIMER.


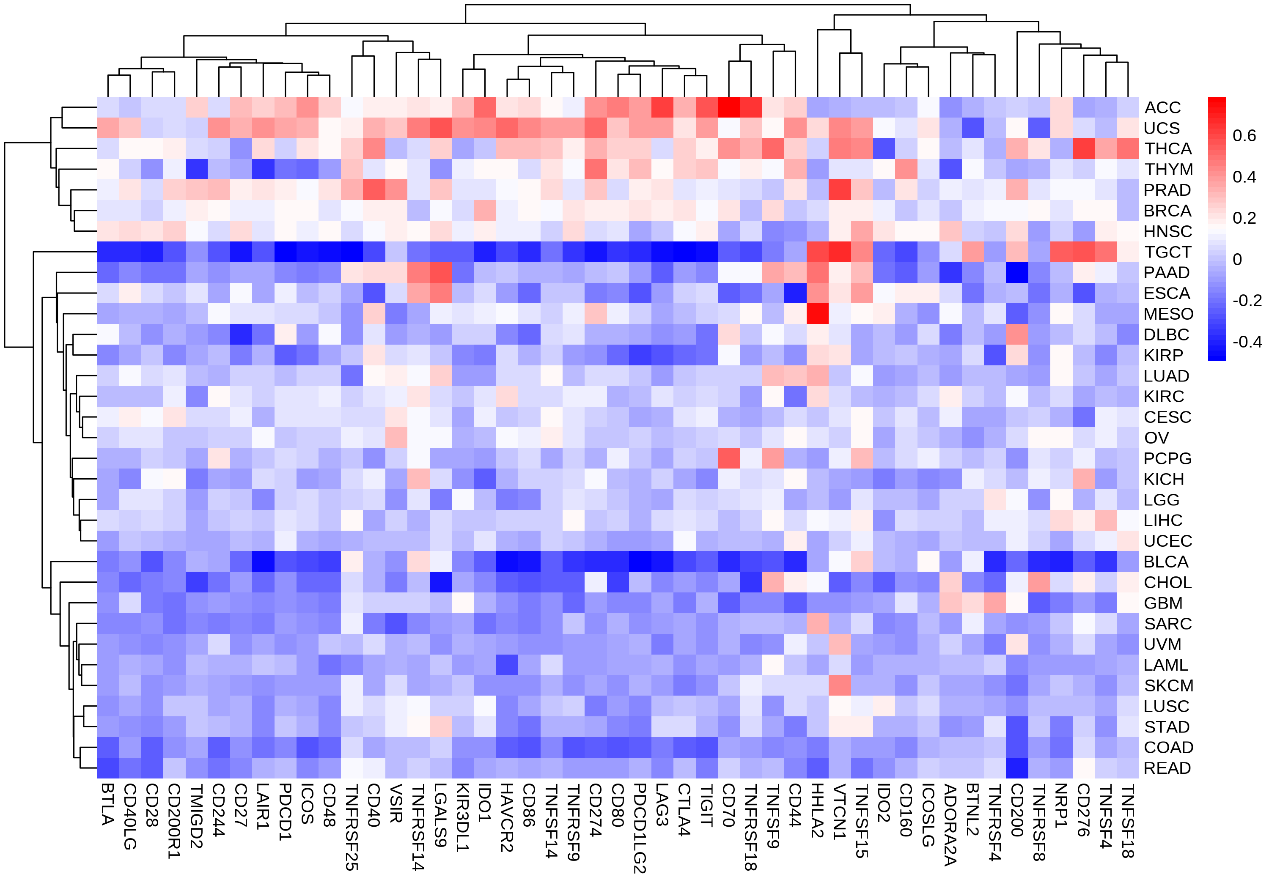


FIGURE S2: The cluster analysis that co-expression of FOXQ1 with immune-related genes in pan-cancer.
